# A Pedestrian Detection Algorithm Based on Score Fusion for Multi-LiDAR Systems

**DOI:** 10.3390/s21041159

**Published:** 2021-02-07

**Authors:** Tao Wu, Jun Hu, Lei Ye, Kai Ding

**Affiliations:** 1 College of Intelligence Science and Technology, National University of Defense Technology, Changsha 410073, China; wutao@nudt.edu.cn (T.W.); hujun17@nudt.edu.cn (J.H.); 2 Science and Technology on Near-Surface Detection Laboratory, Wuxi 214035, China

**Keywords:** pedestrian detection, sliding window, sensor fusion, autonomous vehicles

## Abstract

Pedestrian detection plays an essential role in the navigation system of autonomous vehicles. Multisensor fusion-based approaches are usually used to improve detection performance. In this study, we aimed to develop a score fusion-based pedestrian detection algorithm by integrating the data of two light detection and ranging systems (LiDARs). We first evaluated a two-stage object-detection pipeline for each LiDAR, including object proposal and fine classification. The scores from these two different classifiers were then fused to generate the result using the Bayesian rule. To improve proposal performance, we applied two features: the central points density feature, which acts as a filter to speed up the process and reduce false alarms; and the location feature, including the density distribution and height difference distribution of the point cloud, which describes an object’s profile and location in a sliding window. Extensive experiments tested in KITTI and the self-built dataset show that our method could produce highly accurate pedestrian detection results in real-time. The proposed method not only considers the accuracy and efficiency but also the flexibility for different modalities.

## 1. Introduction

Pedestrian detection and tracking play an essential and significant role in diverse transportation applications [1], such as advanced driver-assistance systems (ADAS), video surveillance systems, and autonomous vehicles. Research in this area is actively ongoing. Many exciting approaches for pedestrian detection have been proposed using cameras [2] or light detection and ranging (LiDAR) [3]. Compared with cameras, LiDARs are becoming more popular due to their ability to generate highly accurate three-dimensional information. Studies on LiDAR-based pedestrian detection have been ongoing for several years; some model-based [4,5] and deep-learning-based [6,7,8,9,10] approaches have been proposed. Besides these, many multi-LiDAR systems aim to overcome the limited vertical resolution and field of view of a single LiDAR. Multi-LiDAR fusion-based approaches usually improve detection performance.

Fusing the measurements of each sensor has become a critical problem to overcome. There are primarily two groups of data fusion strategies: pre-classification and post-classification [11]. Measurement integrations occurring at raw data level or feature level are considered pre-classification fusion. The fusion of information after classification includes: score level, rank level, and decision level [12]. For multi-LiDAR systems, classic approaches working in raw data fusion are well-established. They have thus been extensively used for environment reconstruction [13], moving object [14], and negative obstacles [15] detection. Generally, to integrate different types of information sources, we need feature- or higher-level fusion strategies. Notably, these high-level algorithms have two significant advantages over the raw data-level representation: data compression and noise suppression. Premebia et al. [16] proposed a LiDAR- and vision-based pedestrian detection system with a fusion process occurring at the feature level. More recently, decision fusion methods have attracted more attention for lane [17] and object [18] detection. Typically, these decision-level fusion methods can provide object detection and classification [19] under a probabilistic framework [20].

These kinds of algorithms require the extrinsic calibration of different sensors [21]. Due to the autonomous vehicles navigating in a dynamic environment and LiDAR sensors providing a low data update rate, multi-LiDAR systems need careful spatial and temporal synchronization. Many motion compensation [22] algorithms have been developed to overcome the ego-motion error. However, even after elaborate calibration work, the relative movement between vehicles and pedestrians, the temporal synchronization error, and the phase lock offset between LiDARs can still produce some mismatch between raw LiDAR data.

This motivated our score-level fusion approach for the multi-LiDAR system. We propose a real-time and easy-to-deploy pedestrian detection solution based on two LiDARs. Compared with the rank or decision level, score-level fusion provides more insight into the post-classification process. With this in mind, we first rasterize the point cloud on theh *x*-*y* plane of the LiDAR coordinate system. Then, a 3D sliding window is adopted on the *x*-*y* plane to generate the proposals. We propose two new features to speed up the sliding process and reduce false alarms: the central points density feature and the location feature. These two types of features act as a filter and a coarse classifier, respectively, to reject false positives in the early stage, while the remains can be treated as candidate windows. A fine classifier adopting AdaBoost then performs on seven kinds of geometric features to obtain the scores. After that, we adopt the non-maximum suppression (NMS) process to reduce overlapping windows. The detection results from these two sub-LiDARs are then fused using the Bayesian rule. The comprehensive performance of the proposed method is evaluated by designing several experiments on KITTI and our self-built dataset.

Our proposal has three advantages: (1) it does not need precise time synchronization and motion compensation, (2) it can provide higher precision than a single LiDAR and raw-level fusion of two LiDARs, and (3) it is easily implemented and has a small computational burden. Though we only used two LiDARs to evaluate this algorithm’s efficiency, this framework is flexible and can be quickly extended to more LiDARs or cameras.

The rest of this paper is organized as follows: In Section 2, we introduce some related works. Section 3 provides an overview of the proposed approach. In Section 4, experimental results are presented in two parts. Finally, the conclusions are summarized in Section 5.

## 2. Related Work

LiDAR-based pedestrian detection has been a hot topic in recent years. It is an essential module in the environment perception system of autonomous vehicles.

Inspired by image-based object detection [4,5], some model-based approaches have been proposed. In these approaches, point clouds are firstly segmented based on an unsupervised clustering method, and hand-crafted features are then extracted from the object candidate to train classifiers such as support vector machine (SVM) [23]. The main disadvantage of this approach is the potential incorrect segmentation, including over- and under-segmentation. The quality of the segmentation is vital for the following classification of point clouds.

In recent years, due to their excellent performance, deep learning-based approaches have been widely used. These approaches can be mainly divided into two types. The first type represents the point cloud as 3D voxel grids [8] or 2D orthogonal images by projecting it to the 2D ground plane, and then convolutional neural networks (CNNs) can be applied. BirdNet [10] and TopNet-HighRes [24] both project the 3D point cloud to the bird’s eye view (BEV) and then adopt a deep neural network for detection. The method in Li et al. [7] projects the 3D point cloud to the front-view depth map and applies a 2D CNN on it. Ku et al. [25] and Chen et al. [26] proposed networks that combine features extracted from LIDAR views and RBG images for 3D object detection. The second type involves using the point cloud’s raw coordinates as the input and processing them through an integrated network architecture [27,28]. Complexer-You Only Look Once (YOLO) [29] can achieve real-time 3D object detection with state-of-the-art accuracy. However, the deep-learning-based approaches have a high computational cost and require specific computational devices such as graphical processing units (GPUs), hindering their practical application.

It is generally thought that denser LiDAR points may lead to better detection performance. Therefore, some researchers have tried to fuse data from multiple independent LiDARs. Extensive work has been completed in this area but it primarily concerned raw data fusion methods. Kevin et al. used multiple LiDARs to detect both obstacles and geometric features such as curbs, berms, and shoulders [30]. Mertz et al. [14] proposed a moving object detection system. They fused multiple sensors, including 2D and 3D LiDARs. Negative obstacles considerably influence autonomous vehicle safety because they are difficult to detect at very early stages [31]. Larson et al. presented a negative obstacle detector using a Velodyne HDL-64E [32]. Different from the traditional upright set up on the roof of the vehicle, Shang et al. [15] proposed a novel setup method of two LiDARs mounted with a tilt angle on two sides of the vehicle roof. With an overlap area, the LiDAR data density in front of the vehicle was considerably improved, which is beneficial for detecting negative obstacles.

## 3. The Proposed Approach

**Figure 1:**
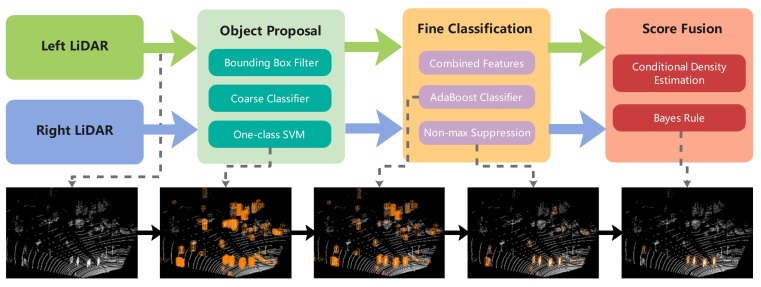
Overview of the proposed approach.

The overall workflow of this study design is summarized in the top half of Figure 1 while the corresponding detection procedures are graphically shown in the lower half. The method consists of three major modules: object proposal, fine classification, and score fusion.

The first module, which performs object candidates, contains a bounding box filter, a coarse classifier, and a one-class SVM. We apply two low-dimensional features in this module to improve the operation efficiency. Fine classification aims to classify further, score the selected sliding window, and eliminate overlapping windows. This module includes combined features computation, AdaBoost classification, and NMS. The last module fuses the information of the left and right LiDARs in the Bayesian framework. We use a parametric method to estimate the match scores’ conditional densities before using the Bayesian rule. A more detailed description of our approach is provided in the following section.

### 3.1. Object Proposal

Object proposal is an effective method to increase the computational efficiency of object detection [33]. The object proposal aims to generate object candidates, which are then passed to an object classifier [34]. Previously constructed methods usually perform the ground segmentation for the original point cloud and then cluster the non-ground points to generate object candidates [35]. However, this usually leads to under-segmentation, especially when groups of people walk together. We adopt an improved sliding window algorithm in this section to overcome this problem, including two stages: a bounding box filter and a coarse classifier.

We firstly discretize the input raw point cloud data into a 2.5D grid map on the *x*-*y* plane with a fixed resolution of 0.1 m × 0.1 m. Each corresponding grid stores all the height information in it. The grid map contains 50 m in front of the vehicle and 25 m on each side. We use a 0.7 m × 0.7 m sliding window to traverse the entire search area with a step size of 0.1 m.

#### 3.1.1. Bounding Box Filter

To speed up the sliding process, we construct a bounding box filter using the following rules: (1) if the central cell is empty, the current window will be bypassed; (2) if the difference between the maximum height and the minimum height of the central cell is within a threshold, the points in the whole window are segmented as pedestrian candidates; and (3) we propose a central points density feature to further filter out the non-pedestrian candidates.

**Figure 2:**
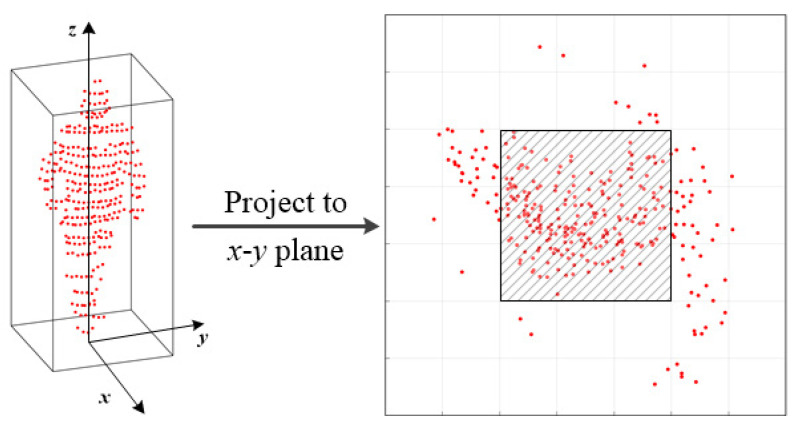
Illustration of the proposed central points density feature.

The central points density feature represents the relative concentration of the middle grids to the sliding window. The schematic of the feature is shown in Figure 2. The left column shows an extracted object candidate. The outer bounding box denotes a sliding window with a length and width of 0.7 m. The sliding window consists of 7×7 grids and the central window contains 3×3 grids.

Let *F* be the value of central points density feature, which is defined as:

(1) $$F=\frac{\sum n_{ij}}{N},\;i,j\in \left\{3,\ldots ,5\right\},$$

where *N* represents the number of points in the sliding window an $n_{ij}$ denotes the number of points falling in the central window. With Equation (2), we can filter out bounding boxes that do not efficiently meet these criteria.

(2) $$\left\{\begin{array}{c} N_{center}>0\\ 0.5<\Delta h<2\\ F>0.35 \end{array}\right.$$

#### 3.1.2. Coarse Classifier

To further improve the computational efficiency, we adopt a coarse classifier after the bounding box filter step. As most pedestrians are upright, it is imperative to consider an ideal bounding box that should keep the target in its center. The extracted point cloud should be complete and avoid including irrelevant surrounding points. We propose a statistical location feature including the density distribution and height difference distribution of the point cloud.

**Figure 3:**
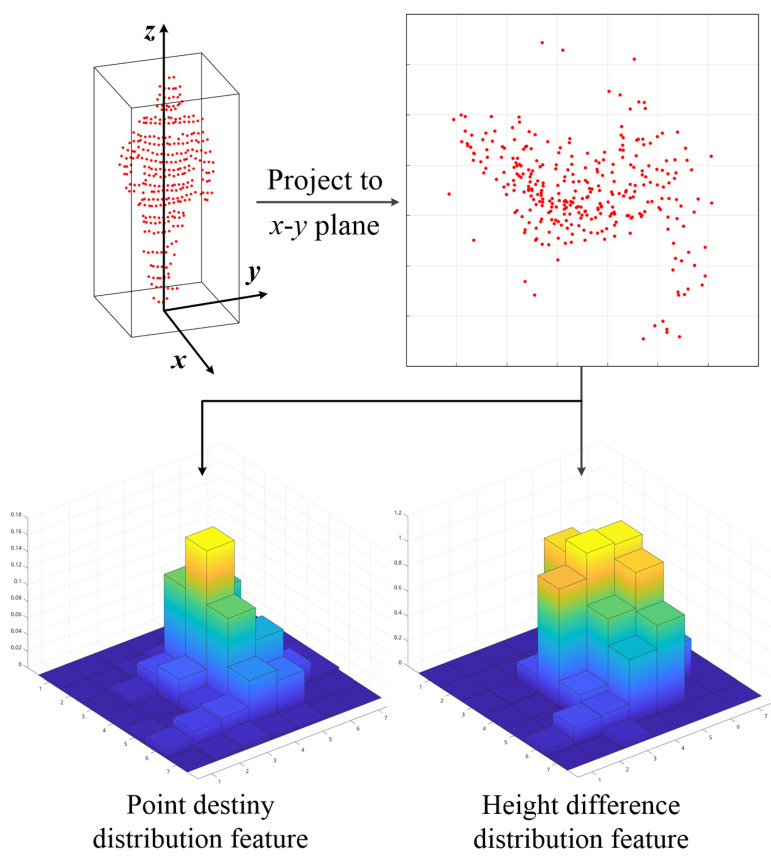
Definition of the proposed location feature.

The schematic of the feature is shown in Figure 3. The same as for the center point density feature, we first project the points in the sliding window into 7×7 grids on the *x*-*y* plane. Then, the element in each grid is calculated using Equation (3).

Let $D_{ij}$ and $\Delta H_{ij}$ be the density and height differences for the points in each grid, respectively; their definitions are:

(3) $$\left\{\begin{array}{c} D_{ij}=\frac{n_{ij}}{N},\;i,j\in \left\{1,\ldots ,7\right\}\\ \Delta H_{ij}=h_{ij_{\max}}-h_{ij_{\min}},\;i,j\in \left\{1,\ldots ,7\right\} \end{array}\right.,$$

where $h_{ij_{\max}}$ and $h_{ij_{\min}}$ indicate the height difference between the highest point and the lowest point in the corresponding grid, respectively.

Until now, we have been trying to finish the object proposal step. It can be considered an anomaly detection problem [36], treating the center position targets as positive samples. Therefore, we then feed the location feature vector into a one-class SVM [37] classifier to generate object candidates.

### 3.2. Fine Classification

We manually label positive samples to train AdaBoost classifiers for each sub-LiDAR. We choose to extract a combined feature, including seven kinds of geometric properties, as shown in Table 1. The set of feature values of each object candidate forms a vector $f=(f_{1},...,f_{7})$. Features $f_{1}$, $f_{2}$, and $f_{3}$ [38] describe the geometric properties of the object point cloud, which can be used to initially classify the point cloud. They are the number of points, the distance from the autonomous vehicle, and the point cloud’s maximum height in the *z* direction, respectively. Features $f_{4}$ and $f_{5}$ are the three-dimensional covariance matrix *C* and its eigenvalues, respectively. The covariance matrix is composed of six independent vectors, and the eigenvalues are arranged in descending order. The matrix *C* is defined as:

(4) $$C=\left(\begin{array}{ccc} \mathrm{cov}(x,x) & \mathrm{cov}(x,y) & \mathrm{cov}(x,z)\\ \mathrm{cov}(y,x) & \mathrm{cov}(y,y) & \mathrm{cov}(y,z)\\ \mathrm{cov}(z,x) & \mathrm{cov}(z,y) & \mathrm{cov}(z,z) \end{array}\right),$$

where:

(5) $$\mathrm{cov}\left(x,y\right)=\frac{\sum_{n=1}^{N}\left(x_{i}-\bar{x}\right)\left(y_{i}-\bar{y}\right)}{n-1}.$$

Feature $f_{6}$ is an inertia tensor matrix [39], which is physically equivalent to the mass in Newtonian mechanics. It describes the overall distribution of the point cloud stably. The matrix *I* is defined as:

(6) $$I=\left(\begin{array}{ccc} I_{xx} & I_{xy} & I_{xz}\\ I_{yx} & I_{yy} & I_{yz}\\ I_{zx} & I_{zy} & I_{zz} \end{array}\right),$$

where:

(7) $$\left\{\begin{array}{c} I_{xx}=\sum_{n=1}^{N}\left(y_{i}^{2}+z_{i}^{2}\right)\\ I_{xy}=I_{yx}=-\sum_{n=1}^{N}x_{i}y_{i} \end{array}\right.,$$

where *x*, *y*, and *z* represent the 3D coordinates of each point, and *N* denotes the number of points of the point cloud.

Feature $f_{7}$ describes the rotational projection statistics [40], which are obtained by rotationally projecting the adjacent points of a feature point onto 2D planes and calculating a set of statistics of the distribution of these projected points.

In the training phase, we adopt a data augmentation strategy to enhance classification performance. We use a mask to randomly remove part of the point cloud at a ratio of 10%, 30%, or 50% to simulate occlusion. During the testing phase, each sub-LiDAR classifier outputs a score within [−100, 100] to show a pedestrian’s likelihood estimation.

We applied an NMS strategy to merge overlapping detections. The traditional NMS approach sorts the bounding boxes according to the detection scores. However, the object with the highest score is not necessarily the best, especially for point clouds. In our experiments, it caused a jitter of the object position and more false alarms in the detection of continuous frames. Therefore, we chose to use the number of points in the bounding box as the sorting criterion. The intersection over union (IoU) between the two object windows was used to judge whether they belong to the same object.

### 3.3. Score Fusion

Kittler et al. developed a theoretical framework for decision-making from multiple classifiers, which are representations derived from the same input source [41]. For the problem of classifying an input *X* into one of *N* possible classes $\left\{y_{1},y_{2},\ldots ,y_{N}\right\}$ based on *M* different classifiers, based on the Bayesian decision theory [42], the input pattern should be assigned to the class $y_{r}$ that maximizes the posterior probability.

(8) $$\begin{array}{cc} \; & \;\mathrm{Assign}\;X\to y_{r}\;\;\mathrm{if}\;\\ \; & P\left(y_{r}|x_{1},\ldots ,x_{R}\right)\geq P\left(y_{k}|x_{1},\ldots ,x_{R}\right) \end{array}$$

In our scenario, we can write:

(9) $$\begin{array}{cc} \; & \;\mathrm{Assign}\;X\to Ped\;\;\mathrm{if}\;\\ \; & P\left(Ped|{Score}_{L},{Score}_{R}\right)\geq P\left(\neg Ped|{Score}_{L},{Score}_{R}\right). \end{array}$$

In Equation (9), $Score_{L}$ and $Score_{R}$ indicate the scores output by the left and right classifiers, respectively; $P(Ped|Score_{L},Score_{R})$ specifies the probability of there being a pedestrian conditioned on the scores from the left and right classifiers.

Currently, there are three broad categories to estimate these posterior probabilities: density-based score fusion, transformation based score fusion, and classifier based score fusion [12]. When the scale of training data is relatively small, the transformation-based score fusion method can be used to achieve classification using sum, max, or min classifier combination rules. Classifier based score fusion combines all scores into a feature and uses a patteren classifier to estimate $P\left(Ped|{Score}_{L},{Score}_{R}\right)$ indirectly. In this study, we use KITTI and self-build data sets, with relatively large amounts of training data, so we choose density-based sore fusion to directly estimate the posterior probability.

The posterior probability can be expressed in terms of conditional joint probability densities using the Bayes rule as follows:

(10) $$\left\{\begin{array}{c} P\left(Ped|{Score}_{L},{Score}_{R}\right)=\frac{P\left({Score}_{L},{Score}_{R}|Ped\right)\cdot P\left(Ped\right)}{P\left({Score}_{L},{Score}_{R}\right)}\\ P\left(\neg Ped|{Score}_{L},{Score}_{R}\right)=\frac{P\left({Score}_{L},{Score}_{R}|\neg Ped\right)\cdot P\left(\neg Ped\right)}{P\left({Score}_{L},{Score}_{R}\right)}. \end{array}\right.$$

Under the assumption of the conditional independence of two classifiers, the conditional joint probability density can be expressed as the product of the marginal conditional densities, i.e.,

(11) $$\left\{\begin{array}{c} P\left({Score}_{L},{Score}_{R}|Ped\right)=P\left({Score}_{L}|Ped\right)\cdot P\left({Score}_{R}|Ped\right)\\ P\left({Score}_{L},{Score}_{R}|\neg Ped\right)=P\left({Score}_{L}|\neg Ped\right)\cdot P\left({Score}_{R}|\neg Ped\right), \end{array}\right.$$

where $P(Score_{L,R}|Ped)$ represents the score distribution of a pedestrian. Substituting Equations (10) and (11) into Equation (9), we obtain:

(12) $$\begin{array}{cc} \; & \;\mathrm{Assign}\;X\to Ped\;\;\mathrm{if}\;\\ \; & \frac{P\left({Score}_{L}|Ped\right)\cdot P\left({Score}_{R}|Ped\right)}{P\left({Score}_{L}|\neg Ped\right)\cdot P\left({Score}_{R}|\neg Ped\right)}\geq \frac{P\left(\neg Ped\right)}{P\left(Ped\right)}=\eta . \end{array}$$

Density estimation generally comes in two ways, by parametric or non-parametric methods [42]. If the form of the density function is assumed to be known, we can use parametric methods to estimate the parameters. k-nearest neighbour (k-NN) or some other data-driven methods do not make any assumption about the density function. In this work, we start by assuming that the likelihood probability follows a Gaussian distribution.

**Figure 4:**
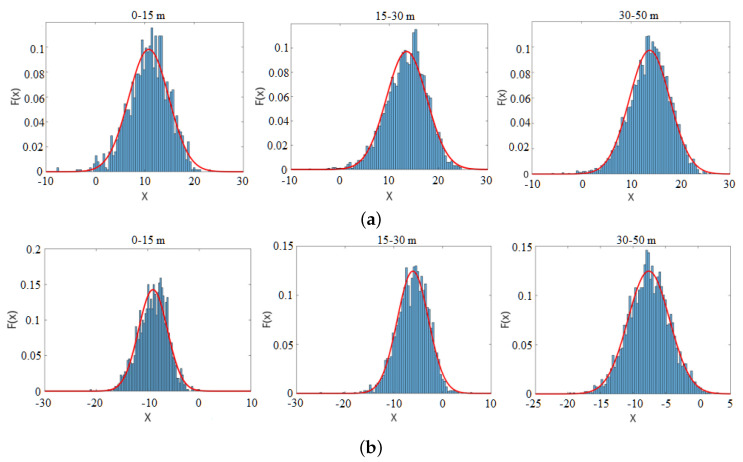
The score distribution of typical samples collected at different distances. The score distributions of (**a**) pedestrians and (**b**) trees.

To verify the Gaussian distribution assumption, we chose pedestrians and trees as two kinds of objects and collected samples at different distances. Figure 4 illustrates the score distribution of these samples collected at different distance ranges. The score histogram is shown as the blue bar. The red line represents the fitted Gaussian distribution curve. The figure shows that the score is similar to the fitted Gaussian curve, thus verifying the Gaussian distribution assumption.

**Figure 5:**
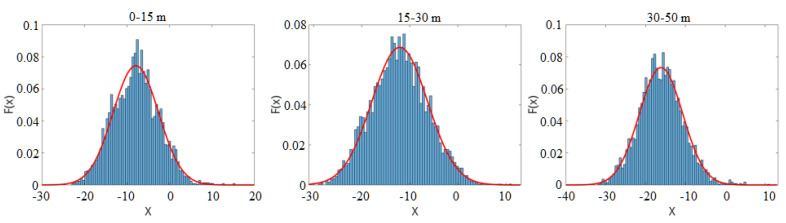
The score distribution of the negative samples collected at different distances.

To analyze the negative samples’ score distribution, 4200 non-pedestrian samples, including cars, trees, shrubs, fences, etc., were collected at different distances. The score distribution of the overall negative samples is shown in Figure 5. The figure illustrates that the score histogram is also similar to the fitted Gaussian curve. $P(Score_{L,R}|Ped)$ and $P(Score_{L,R}|\neg Ped)$ can be described as:

(13) $$\left\{\begin{array}{c} P\left(Score_{L,R}|Ped\right)=\frac{1}{\sqrt{2\pi }\sigma_{pos}}\exp\left(-\frac{{\left(Score_{L,R}-\mu_{pos}\right)}^{2}}{2\sigma_{pos}^{2}}\right)\\ P\left(Score_{L,R}|\neg Ped\right)=\frac{1}{\sqrt{2\pi }\sigma_{neg}}\exp\left(-\frac{{\left(Score_{L,R}-\mu_{neg}\right)}^{2}}{2\sigma_{neg}^{2}}\right), \end{array}\right.$$

where $\left\{\mu_{pos},\sigma_{pos}\;\mu_{neg},\sigma_{neg}\right\}$ can be estimated from the training data.

Therefore, the score fusion algorithm is applied as follows:

1. Each testing sample is given a $Score_{L}$ and $Score_{R}$ by the classifiers of two sub-LiDARs.
2. The scores of positive and negative samples are sent to the Gaussian distribution function.
3. According to Equation (12), by comparing the posterior probability, the sample is classified as a pedestrian or not.

## 4. Experimental Results

To evaluate the effectiveness of the proposed algorithms, extensive experiments were conducted. First, we evaluated our pedestrian detection approach without score fusion on the KITTI 3D object detection benchmark [43], which consists of 7481 training frames and 7518 test frames from a Velodyne 64E LiDAR. After splitting the training data into a training set (3712 frames) and a validation set (3769 frames) [26], we compared our approach with state-of-the-art pedestrian detection methods. The models were all trained on the training split and evaluated on the test split and the validation split.

Then, we evaluated the whole pipeline on our self-build data sets. Our experimental platform included a laptop equipped with a quad-core 2.3 GHz Intel i5 CPU and 8 GB of RAM.

### 4.1. Experiment on KITTI Data Set

**Figure 6:**
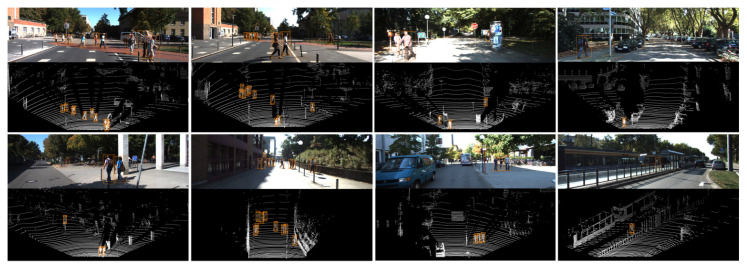
Visualization of our results on the KITTI test set. The detected pedestrians are indicated with orange 3D bounding boxes in the LiDAR view. The 3D bounding boxes are projected onto the corresponding image in the upper row.

Our approach was evaluated on 3D detection and BEV detection on the KITTI’s official test server. Figure 6 shows some examples of the detection results. The results were calculated according to the easy, moderate, and hard difficulty levels provided by KITTI. As shown in Table 2, our proposed method significantly outperformed previous state-of-the-art methods. Among them, AVOD [25] and Complexer-YOLO [29] use both point clouds and RGB images. BirdNet [10] and TopNet-HighRes [24] are LiDAR-only methods using convolutional neural networks (CNNs).

Our approach, based on traditional models and only taking point clouds as the input, produced more competitive results than AVOD in BEV detection and outperformed the other methods by large margins on all difficulty levels in both tasks. Our approach only requires about 0.026 s runtime per frame on a quad-core CPU. This is more than twice as fast as AVOD and Complex-YOLO and four times faster than BirdNet.

In NMS, we compared the differences in the scores generated using the proposed location features and the final classifier. The influences of each module on the detection performance was analyzed by only removing the specific part and keeping all other parts unchanged. The results illustrated in Table 3 show that by adopting our proposed filter, the processing time is significantly reduced (from 96 to 39 ms and from 32 ms to 26 ms), and the detection performance is improved. This result demonstrates that the proposed filter is effective for accurately filtering out non-pedestrian proposals.

Generating scores for proposals using the location feature in NMS could further reduce computation time while maintaining similar performance compared to directly using the final classifier.

### 4.2. Setup of Self-Built Data Set

**Figure 7:**
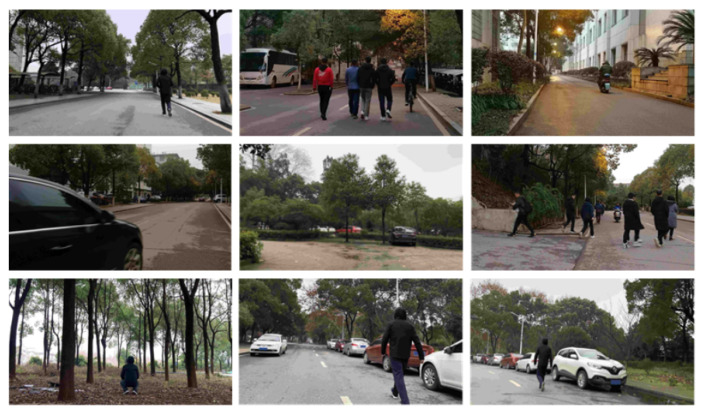
Examples of test scenarios in the experiment.

Two self-built data sets were prepared for the evaluation. Data set I contains five people walking in front of a parked vehicle. The measurement range is from 0 to 50 m. The total number of frames was 1676. We manually labeled the window of each pedestrian as positive samples. Details of the labeled samples are listed in Table 4. Data set II was collected in the real road environment. The training and evaluating data were extracted on different road segments. The measurement range was also up to 50 m. Details of the samples are listed in Table 5 and some examples of different test scenarios are shown in Figure 7.

### 4.3. Comparison of Object Proposal

As a basis for detection, we first tested the object proposal algorithms using data set I. To perform a more accurate evaluation, we used the following criteria:

1. Over-segmentation: clusters that contain fewer than 70% of the ground truth points;
2. Under-segmentation: clusters that contain more than one object or the object is lost.

**Figure 8:**
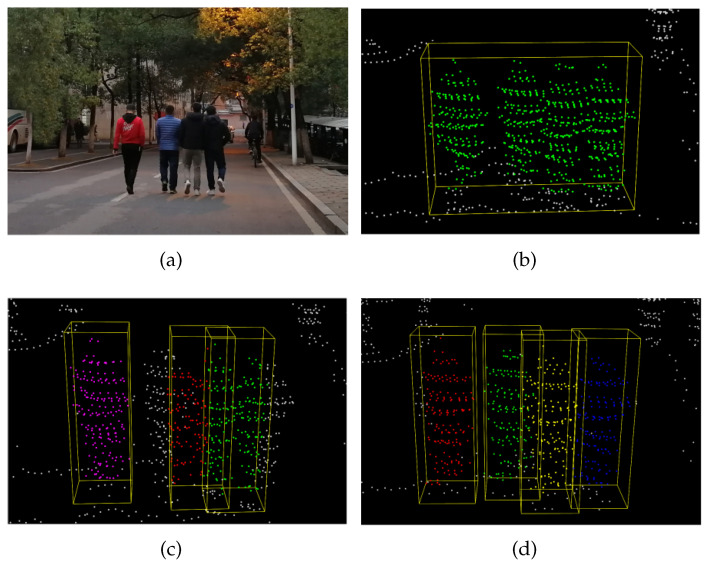
An example of the better pedestrian proposal results produced by the proposed approach. (**a**) A scenario from the experiment; (**b**) the output of the clustering algorithm. The four pedestrians walking side-by-side are clustered together. (**c**) The output of the normal sliding window algorithm. Note that the three pedestrians on the right are mistakenly segmented. (**d**) The output of the proposed algorithm: the four pedestrians are correctly segmented. The color of the different clusters is randomly chosen by the algorithms.

The profile in Table 6 illustrates the results of the quantitative analysis. The proposed method performed better on all indicators compared to the other approaches. Clustering performance was poor due to a large number of under-segmentation errors. Compared to the typical sliding window algorithm, the proposed method’s recall increased by an average of 0.1 within 30 m. Note that as the distance increases, the segmentation accuracy decreases due to the sparsity of the point cloud, whereas our approach maintains relatively high performance. An example of the pedestrian proposal performance of the different algorithms is shown in Figure 8. The result showed that our approach can work well in complex scenarios.

### 4.4. Comparison of Different Classifiers

In this section, the performance of different classification algorithms is evaluated. In traditional methods [13,14,15], the raw data of two LiDARs are fused as a whole point cloud, and a fixed threshold is set for classification.

For a more detailed evaluation, all testing samples were divided into three categories: 0–15, 15–30, and 30–50 m. To eliminate the model’s impact on the results, we used three different models as the classifiers: AdaBoost, SVM, and PointNet. The same model adopted the same parameter settings. The parameters of PointNet were as follows: batch size = 32, max. epoch = 250, learning rate = 0.001, and the optimizer was Adam.

**Figure 9:**
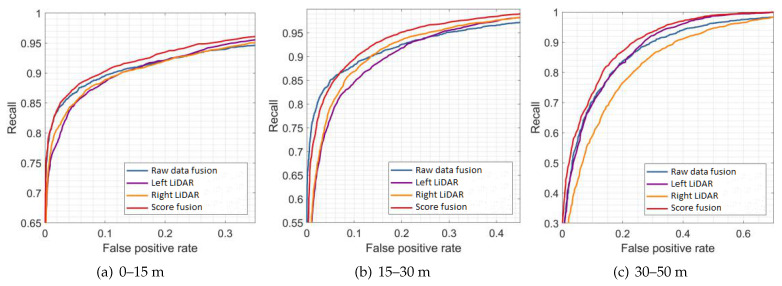
Results of evaluations at different ranges (AdaBoost).

**Figure 10:**
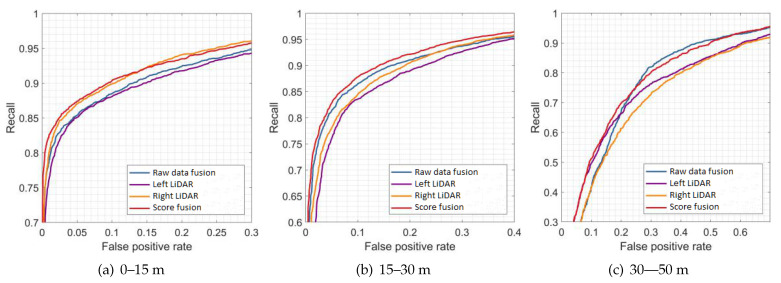
Results of evaluations at different ranges (SVM).

**Figure 11:**
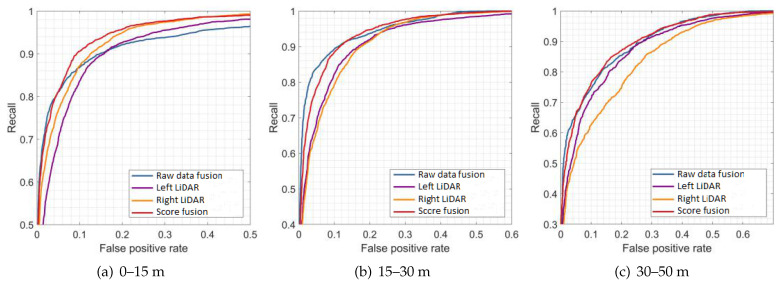
Results of evaluations at different ranges (PointNet).

Figure 9, Figure 10 and Figure 11 show the results of the evaluation with different classifiers at different ranges, which are presented as the receiver operating characteristic (ROC) curve. The output of the raw data fusion is shown as a reference. The results of the two sub-LiDARs are also presented. Table 7 lists their area under the curve (AUC).

The performance of the two sub-LiDARs is generally lower than that of raw data fusion. However, by fusing the results of two sub-LiDARs, the proposed approach performed better than the reference, even at ranges between 30 and 50 m, where the density of the point cloud significantly decreases. It is considered that the point cloud of the sub-LiDARs is sufficiently dense, and the score fusion algorithm can overcome the detection error of a single LiDAR.

**Figure 12:**
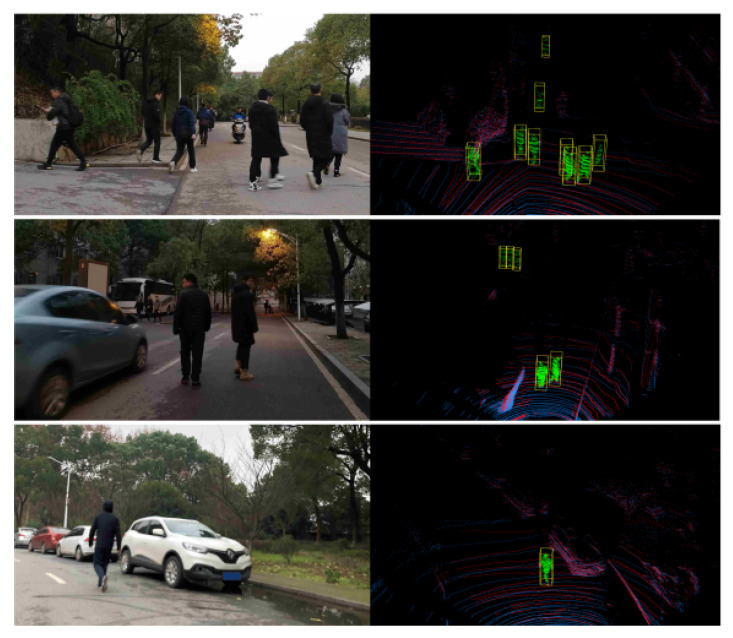
Typical experimental results on real road scenarios.

Some typical experimental results are shown in Figure 12: the four pedestrians on the right side of the first-row image are correctly detected. Three pedestrians walking close-by are also successfully detected, as shown in the second row of Figure 12.

### 4.5. Comparison of Processing Speed

For algorithms applied to autonomous vehicles, another important performance criterion is the processing speed. To evaluate the proposed algorithms’ computational efficiency, we tested their runtime on 300 continuous data frames. The computational device used for the proposed method contained an Intel Core-i5 CPU and 8 GB RAM. PointNet was evaluated with a GTX 1060 GPU.

The processing times of the different object proposal algorithms are shown in Table 8. The proposed improved sliding window algorithm is the fastest, and its computation time is approximately reduced by 15 ms compared to the clustering algorithm.

The processing times of different pedestrian detection algorithms are shown in Table 9. The object proposal algorithm used here is the improved sliding window algorithm proposed. The raw data fusion method based on a fixed threshold has the fastest average calculation time among the detection algorithms. The proposed algorithm requires slightly more calculation time than the raw data fusion method. PointNet is more computationally intensive and time-consuming. In general, the proposed algorithm’s average processing time is less than 30 ms, which meets the real-time requirements of autonomous vehicles.

## 5. Conclusions

This paper proposed a pedestrian detection algorithm based on score fusion, achieving a reasonable balance between accuracy and efficiency. The real-time performance of sensing algorithms is a critical issue for autonomous vehicles. Suppose an autonomous vehicle cruising on a street at a speed of 60 km/h; during the 0.026 s runtime of our approach, the vehicle will travel about 0.5 m. Meanwhile, considering the maximum detection distance is more than 40 m, our approach’s real-time performance meets the requirements.

The experimental results demonstrated that our approach can achieve higher accuracies than traditional raw data fusion algorithms in most cases. The proposed framework’s flexibility allows for different kinds of classification algorithms to be employed before the fusion process. However, when the pedestrian is partially occluded, the detection accuracies obviously declined. Harsh environments, especially in specific weather conditions such as rainfall, snowfall, and particles in the air, have a definite impact on algorithm performance.

In future work, we plan to improve our approach in two aspects: we intend to improve the classifier to increase detection performance, such as by adopting more discriminative features or combining them with lightweight neural networks; and we will try to use different types and quantities of classifiers from various sensors under this framework.

## Figures and Tables

**Table 1:** Description of features.

No.	Description	Dimension
$f_{1}$	Number of points	1
$f_{2}$	Distance to the object	1
$f_{3}$	Maximum height difference	1
$f_{4}$	Three-dimensional covariance matrix	6
$f_{5}$	Three-dimensional covariance matrix eigenvalue	3
$f_{6}$	The normalized moment of inertia tensor	6
$f_{7}$	Rotational projection statistics	135

**Table 2:** Evaluation on KITTI test set for pedestrians.

Method	3D Detection AP (%)			BEV Detection AP (%)			Times (s)
	Easy	Moderate	Hard	Easy	Moderate	Hard	
AVOD [25]	36.10	27.86	25.76	42.58	33.57	30.14	0.08
Complex-YOLO [29]	17.60	13.96	12.70	21.42	18.26	17.06	0.06
BirdNet [10]	12.25	8.99	8.06	20.73	15.80	14.59	0.11
TopNet-HighRes [24]	10.40	6.92	6.63	19.43	13.50	11.93	0.10
Ours	33.75	26.64	23.34	49.27	37.96	33.83	0.026

**Table 3:** Performance on KITTI validation set for pedestrians by adopting different modules.

Filter	NMS		3D Detection AP (%)			BEV Detection AP (%)			Times (s)
	Location Feature	Classifier Score	Easy	Moderate	Hard	Easy	Moderate	Hard	
*√*	*√*		47.54	43.72	37.49	65.03	59.15	51.38	0.026
*√*		*√*	49.77	45.53	39.20	65.30	59.07	51.46	0.039
	*√*		47.41	43.66	37.47	64.93	58.95	51.30	0.032
		*√*	49.40	45.18	38.78	64.25	58.29	50.57	0.096

**Table 4:** Conditions for object proposal algorithms evaluation.

Description	Total	0–15 m	15–30 m	30–50 m
Evaluation data	8380	2828	3392	2160

**Table 5:** Conditions for pedestrian detection algorithms evaluation.

Description	Total	Positive	Negative
Training data	7931	2170	5761
Evaluation data	24,736	9245	15,491

**Table 6:** Comparison of the object proposal algorithms.

Method	0–15 m			15–30 m			30–50 m		
	Over (Over Is Over-Segmentation Rate)	Under (Under Is under-Segmentation Rate)	Recall	Over	Under	Recall	Over	Under	Recall
Clustering	0.0007	0.4975	0.5018	0	0.9366	0.0634	0	0.9681	0.0319
Normal sliding window	0.0601	0.0704	0.8695	0.0183	0.0949	0.8868	0.0125	0.1481	0.8394
This Paper	0.0014	0.0032	0.9954	0.0029	0.0180	0.9791	0.0106	0.1130	0.8764

**Table 7:** Comparison of the area under the curve (AUC) of the pedestrian detection algorithms.

Method	0–15 m			15–30 m			30–50 m		
	Ada.	SVM	PointNet	Ada.	SVM	PointNet	Ada.	SVM	PointNet
Raw data fusion	0.9521	0.9562	0.9467	0.9569	0.9432	0.9644	0.8983	0.8077	0.9246
Left LiDAR	0.9543	0.9543	0.9399	0.9484	0.9294	0.9408	0.9082	0.7924	0.9082
Right LiDAR	0.9510	0.9648	0.9570	0.9526	0.9363	0.9407	0.8648	0.7682	0.8803
This paper	0.9600	0.9652	0.9644	0.9659	0.9494	0.9602	0.9235	0.8185	0.9255

**Table 8:** Comparison of the processing time of the object proposal algorithms.

Method	Average Computing Time (ms)
Clustering	25.65
Normal sliding window	10.71
This paper	10.28

**Table 9:** Comparison of different pedestrian detection algorithms in terms of processing time.

Method	Average Computing Time (ms)
Raw data fusion	23.24
PointNet	56.53
This paper	25.80

## Data Availability

The data presented in Section 4.1 are openly available in KITTI dataset at http://www.cvlibs.net/datasets/kitti/raw_data.php. The data that support the findings in Section 4.2 are available from the corresponding author, L.Y., upon reasonable request.
